# TORCH screening used appropriately in China?─three years results from a teaching hospital in northwest China

**DOI:** 10.1186/s12884-019-2642-7

**Published:** 2019-12-09

**Authors:** Lin-Chuan Wang, Fang Yan, Jing-Xiong Ruan, Yao Xiao, Yan Yu

**Affiliations:** 1 The First Affiliated Hospital of Xi’an Jiaotong University, Xi’an, Shaanxi Province China; 2 Xi’an NO.3 Hospital, Xi’an, Shaanxi Province China; 3 Honghui Hospital, Xi’an Jiaotong University, Xi’an, Shaanxi Province China

**Keywords:** *Toxoplasma gondii*, Rubella virus, Cytomegalovirus, Herpes simplex virus

## Abstract

**Objectives:**

TORCH infections caused by *Toxoplasma gondii* (TOX), rubella virus (RV), cytomegalovirus (CMV) and herpes simplex virus 1,2 (HSV-1,2) are associated with congenital anomalies. The study aimed to analyze the characteristics of TORCH screening in reproductive age women.

**Methods:**

A total of 18,104 women (2015–2017) from a teaching hospital in Xi’an, China, were enrolled in the study. The characteristics of TORCH screening, i.e., the application of TORCH test, the seroprevalence, the impact of age, periods of gestation and woman with bad obstetric history (BOH) on the serological data were investigated.

**Results:**

In the study, 319 women (1.76%) performed dynamic TORCH test. 51.66, 20.44 and 3.83% of the population did the test in the pre-gestation period, the first and third trimester, respectively. Quite a few pre-gestation women (29.74%) ignored screening of IgG antibodies. The overall IgG/IgM seropositvity of TOX, RV, CMV, HSV-1 and HSV-2 was 4.35%/0.35, 90%/0.63, 96.79%/0.97, 81.11%/0.14 and 6.1%/0.19%, respectively. The age-specific distributions and periods of gestation had no significant effect on the seroprevalence of TORCH agents, *p*>0.05. However, BOH was significantly associated with higher seropositvity of IgM (RV, CMV, HSV-1 and HSV-2) and IgG (CMV and HSV-1) antibodies, *p* < 0.05.

**Conclusion:**

In Xi’an region, more attentions should be paid to TOX, CMV, HSV-2 and the women with BOH for TORCH screening. Meanwhile, a greater emphasis needs to be placed on TORCH test used inappropriately in China.

## Introduction

TORCH infections, which also called perinatal or congenital infections, are caused by serial organisms during pregnancy with an acronym TORCH, which named *Toxoplasma gondii* (TOX), Other (hepatitis viruses, parvovirus, human immunodeficiency virus, Epstein-Barr virus, syphilis), Rubella virus (RV), Cytomegalovirus (CMV), and Herpes Simplex Virus (HSV) [1–3]. The diagnosis and management of other TORCH infections, i.e., hepatitis viruses, syphilis and human immunodeficiency virus, are clear and highly efficacious. However, most of the TORCH infections, such as TOX, RV, CMV and HSV, which are easily overlooked and difficult to diagnose due to be often asymptomatic and relatively low virulence, may lead to serious fetal consequences, i.e., abortions, intrauterine foetal deaths, congenital malformations [4–6]. Therefore, early recognition these TORCH infections are important for treatment and prevention strategies to avoid adverse fetal outcomes.

Up to present, only CMV and HSV-2 PCR tests were approved for clinical practice by the China Food and Drug Administration (CFDA). Therefore, the serological evidences through detection of IgM and IgG antibodies against TORCH agents are the preferred approaches for identification of these infections in China. The present study was carried out to investigate the application and epidemiological characteristics of TOX, RV, CMV and HSV screening in reproductive age women from Xi’an region, China.

## Materials and methods

### Study population

The retrospective study was conducted from January 2015 to December 2017 at the First Affiliated Hospital of Xi’an Jiaotong University. A total of 18,104 reproductive age women with TORCH screening were included. The median age was 29 years (range: 20–44 years). Patients with bad obstetric history (BOH) in the study were those with history of previous unfavourable foetal outcome in terms of two or more abortions, infertility, intrauterine growth retardation, intrauterine foetal death, still birth, early neonatal death and congenital anomalies. The data used in the study were obtained from the Laboratory Information System (LIS) of the First Affiliated Hospital of Xi’an Jiaotong University.

### Laboratory tests

#### lgM and lgG antibodies of TORCH agents detection

**Fig. 1:**
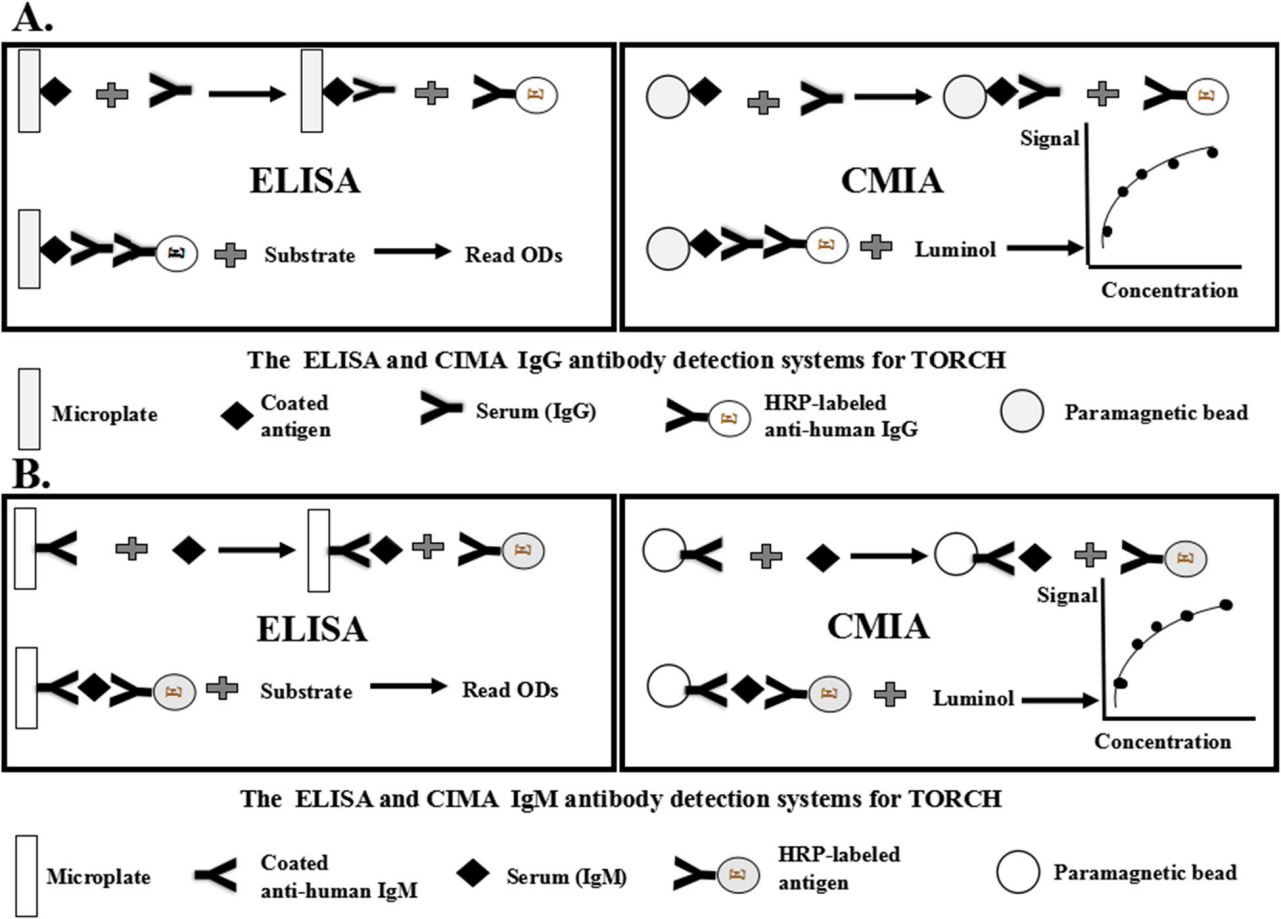
The principle of ELISA and CMIA for (**a**) IgG detection systems of TORCH and (**b**) IgM detection systems of TORCH

The Enzyme-Linked Immunosorbent Assay (ELISA) and Chemiluminesent Micropaticle Immuno Assay (CMIA) were applied to IgM and IgG antibodies detection for TORCH agents before and after 2017, respectively. The commercial ELISA and CMIA kits (Autobio Diagnostics Co., Ltd., Zhengzhou, China) were based on two principles, i.e., the indirect principle for IgG antibody (Fig. 1a) and capture principle for IgM antibody (Fig. 1b). The sera were tested using the Addcare Asp 150/8 analyzor (Addcare Bio-Tech Co., Ltd., Yantai, China) for ELISA and Autolumo A2000 analyzor (Autobio Diagnostics Co., Ltd., Zhengzhou, China) for CMIA according to the manufacturer’s instructions and interpretations.

#### PCR test

Quantification of CMV-DNA and HSV-2-DNA was performed on the DA7600 RealTi*m*e amplification fluorescence detection System (DaAn Gene Co., Ltd., ZhongShan, China) using specific primers. The boiling method (100 °C for 10 min) was used to extract DNA. The PCR parameter was: Pre-denaturation at 93 °C for 2 min, then 93 °C for 45 s → 55 °C for 60s → 10 cycles; 93 °C for 30s → 55 °C for 45 s → 30 cycles. The lower limit of detection is approximately 50 copies/mL.

### Statistical analysis

Statistical analyses were performed by SPSS13.0 (serial number 5026743; SPSS Inc., Chicago, Illinois, USA). Categorical variables were compared using the Fisher exact test. A *p*-value < 0.05 was considered to be statistically significant.

## Results

### The epidemiological characteristics and application of TORCH test

During the study period, 18,104 reproductive age women (20–44 years) were included. The test numbers in 2015, 2016 and 2017 were 4953, 6387 and 6764, respectively. The age-specific distributions of the population were 14.69% for 20–25 years, 53.88% for 26–30 years and 31.43% for ≥31 years. The habitual abortion, infertility and spontaneous abortion accounted for the majority of the women with bad obstetric history (BOH) (56.89, 27.77 and 12.46%, respectively).

In the study, 51.66, 20.44, 24.08 and 3.83% of the women performed TORCH screening in the pre-gestation period, the first, second and third trimester, respectively. The woman attached importance to both IgM and IgG antibodies of TORCH agents in the first and second trimester, with a proportion of 89.11 and 80.06%. However, less than half of the women in the third trimester (44.30%) and with BOH (40.74%) tested both IgM and IgG antibodies. Meanwhile, 29.74% of the pre-gestation women and 18.94% of pregnant women only screened IgM antibody. With age increased, the attention to IgG antibody test decreased, Table 1.

**Table 1:** The epidemiological characteristics and application of TORCH test

Characteristics	No	Application of TORCH test		Others Characteristics	No
		Both IgM and IgG: n(%)	Only IgM: n		
Year				The Distributions of BOH	
2015	4953	4937 (99.68)	16	Infertility	760
2016	6387	4030 (63.10)	2357	Intrauterine foetal death	37
2017	6764	4699 (69.47)	2065	Intrauterine growth retardaion	7
Age				Still birth	18
20~25 years	2659	2082 (78.30)	577	Habitual abortion	1557
26~30 years	9755	7438 (76.25)	2317	Spontaneous abortion	341
≥ 31 years	5690	4146 (72.86)	1544	Missed abortion	4
Before pregnancy	9352	6571 (70.26)	2781	Fetal congenital malformation	13
Periods of gestation	8752	7094 (81.06)	1658	Serologic test	
First trimester	3700	3297 (89.11)	403	Only one time	17,785
Second trimester	4359	3490 (80.06)	869	Two or more times▲	319
Third trimester	693	307 (44.30)	386	PCR test	
BOH group	2737	1115 (40.74)	1622	CMV-DNA test^a^	59
Normal control group	15,367	12,599 (81.99)	2768	HSV-2-DNA test^b^	5

Note: ▲In the first serologic test, 29 cases were seropositive to only IgM antibody (4 for TOX, 2 for RV, 17 for CMV, 2 for HSV-1 and 4 for HSV-2); ^a^45 were serum, 14 were urine;^b^1 was serum and 4 were secretion

### Prevalence of anti-TORCH agents

**Fig. 2:**
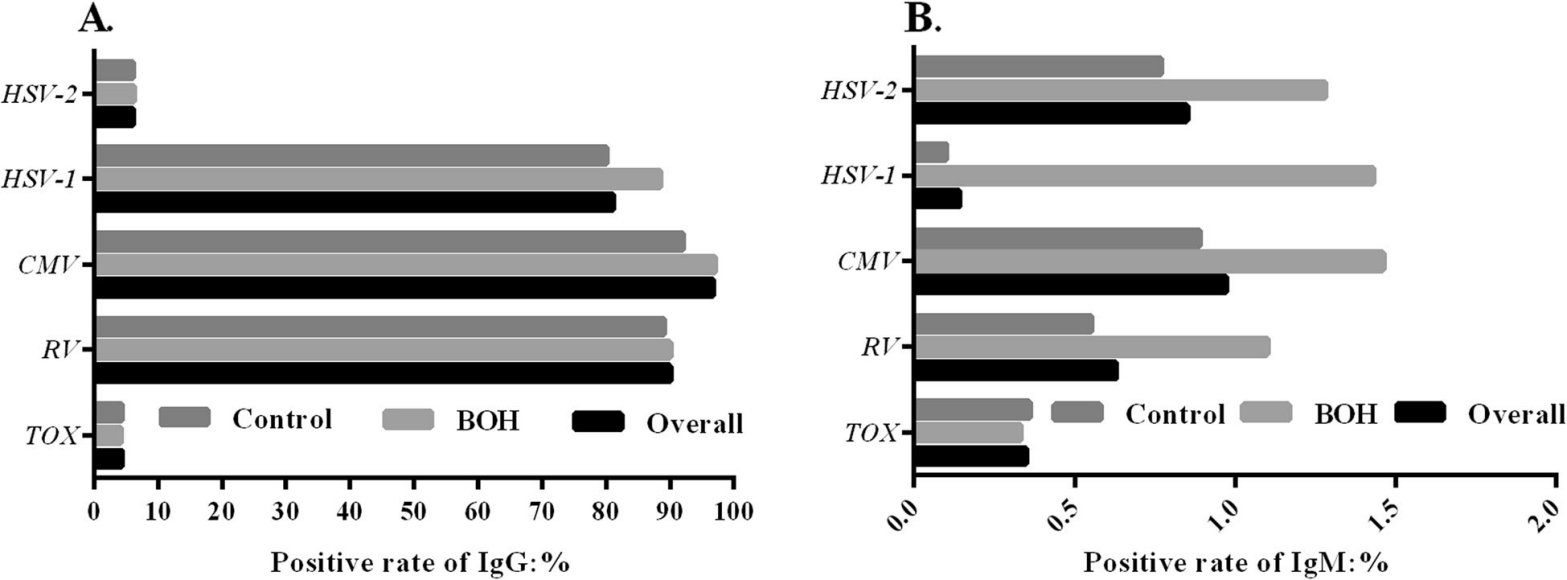
The positive rates of antibodies against TOX, RV, CMV, HSV-1 and HSV-2 in BOH group, normal control group and overall population. **a** IgG; **b** IgM

In our study, the overall seropositvity of TOX, RV, CMV, HSV-1 and HSV-2 was 4.34, 90, 96.79, 81.11 and 6.1% for IgG antibodies whereas it was only 0.35, 0.63, 0.97, 0.14 and 0.85% for IgM antibodies, respectively. In contrast to 2015 and 2016 (ELISA), the IgG seropositvity for TOX, RV, HSV-1 significant decrease and the IgM seropositvity for TOX, RV and HSV-2 significant increase were observed in 2017 (CMIA), *p* < 0.05. The age-specific distributions and periods of gestation had no significant effect on the seropositvity of TORCH agents, *p*>0.05. However, the seropositvity of IgG antibodies (CMV and HSV-1, Fig. 2a) and IgM (RV, CMV, HSV-1 and HSV-2, Fig. 2b) in BOH group were significantly higher than that in normal control group, *p* < 0.05, Table 2.

**Table 2:** The seropositive results of TORCH agents

	TOX: n (%)			RV: n (%)			CMV: n (%)			HSV-1: n (%)			HSV-2: n (%)		
	Ig G	IgM	Both	Ig G	IgM	Both	Ig G	IgM	Both	Ig G	IgM	Both	Ig G	IgM	Both
Overall	594 (4.35)	64 (0.35)	6 (0.04)	12,299 (90.00)	114 (0.63)	75 (0.55)	13,227 (96.79)	176 (0.97)	138 (1.01)	11,084 (81.11)	26 (0.14)	26 (0.19)	834 (6.10)	153 (0.85)	39 (0.29)
Year															
2015	237 (4.8)	7 (0.14)	3 (0.06)	4561 (92.38)	24 (0.48)	22 (0.45)	4784 (96.9)	16 (0.32)	16 (0.32)	4068 (82.4)	12 (0.24)	12 (0.24)	295 (5.98)	39 (0.79)	11 (0.22)
2016	180 (4.47)	9 (0.14)	0 (0)	3652 (90.62)	23 (0.36)	16 (0.39)	3898 (96.72)	18 (0.28)	18 (0.45)	3311 (82.16)	14 (0.22)	12 (0.19)	237 (5.88)	35 (0.55)	8 (0.2)
2017	177 (3.77)	48 (0.71)	3 (0.06)	4086 (86.95)	67 (0.99)	37 (0.79)	4545 (96.72)	142 (2.1)	104 (2.21)	3705 (78.85)	2 (0.03)	2 (0.04)	302 (6.43)	79 (1.69)	20 (0.43)
*p*-value	0.041	0.000	0.284	0.000	0.000	0.023	0.852	0.000	0.000	0.000	0.005	0.006	0.510	0.000	0.108
Maternal age															
20~25 years	89 (4.27)	13 (0.49)	2 (0.1)	1870 (89.82)	21 (0.79)	16 (0.77)	2007 (96.4)	20 (0.75)	16 (0.77)	1676 (80.5)	5 (0.19)	3 (0.14)	130 (6.24)	30 (1.13)	11 (0.53)
26~29 years	301 (4.05)	33 (0.34)	2 (0.03)	6706 (90.16)	56 (0.57)	40 (0.54)	7195 (96.73)	98 (1.0)	75 (1.01)	6038 (81.18)	13 (0.13)	13 (0.17)	446 (6.00)	72 (0.74)	16 (0.22)
≥ 30 years	204 (4.92)	18 (0.32)	2 (0.05)	3723 (89.80)	37 (0.65)	19 (0.46)	4025 (97.08)	58 (1.02)	47 (1.13)	3370 (81.28)	10 (0.18)	10 (0.24)	258 (6.22)	51 (0.9)	12 (0.29)
*p*-value	0.086	0.434	0.407	0.789	0.447	0.289	0.326	0.455	0.397	0.737	0.696	0.64	0.850	0.132	0.06
Period															
Before pregnancy	295 (4.49)	30 (0.32)	3 (0.03)	5910 (89.94)	68 (0.73)	40 (0.61)	6343 (96.53)	78 (0.83)	52 (0.79)	5297 (80.61)	17 (0.18)	6 (0.09)	416 (6.33)	86 (0.92)	16 (0.24)
First trimester	132 (4.0)	14 (0.38)	1 (0.03)	2970 (90.08)	21 (0.57)	20 (0.61)	3194 (96.88)	39 (1.05)	35 (1.06)	2672 (81.04)	5 (0.14)	5 (0.15)	174 (5.28)	25 (0.68)	11 (0.33)
Second trimester	153 (4.38)	17 (0.39)	1 (0.03)	3143 (90.06)	18 (0.41)	14 (0.4)	3391 (97.16)	52 (1.19)	45 (1.29)	2875 (82.38)	5 (0.11)	4 (0.11)	219 (6.28)	39 (0.89)	10 (0.29)
Third trimester	14 (4.56)	3 (0.43)	1 (0.32)	276 (89.90)	7 (1.01)	1 (0.33)	299 (97.39)	7 (1.01)	6 (1.95)	240 (78.18)	1 (0.14)	1 (0.33)	25 (8.14)	3 (0.43)	2 (0.65)
*p*-value	0.729	0.888	0.117	0.996	0.096	0.511	0.329	0.228	0.034	0.091	0.803	0.603	0.074	0.331	0.548
Group															
BOH	47 (4.22)	9 (0.33)	1 (0.09)	1004 (90.04)	30 (1.1)	6 (0.54)	1082 (97.04)	40 (1.46)	19 (1.70)	987 (88.52)	16 (1.43)	15 (1.35)	69 (6.19)	35 (1.28)	9 (0.81)
Normal control	549 (4.36)	55 (0.36)	5 (0.04)	11,295 (89.65)	84 (0.55)	69 (0.55)	11,592 (92.01)	136 (0.89)	121 (0.96)	10,097 (80.14)	12 (0.10)	11 (0.09)	765 (6.07)	118 (0.77)	30 (0.24)
*p*-value	0.871	0.492	0.399	0.716	0.002	1.000	0.000	0.006	0.028	0.000	0.000	0.000	0.851	0.010	0.003

### Results of serial serologic tests and PCR

In the study, 319 women (1.76%) performed two or more times serologic tests. Among 29 cases who were seropositive to only IgM antibody in the first screening, 6 cases had IgG antibody seroconversion (1 for TOX and 5 for CMV). The persistence and disappearance of IgM antibody was found in 20 and 3 cases, respectively. Additionally, 64 women performed both serological and PCR tests for CMV (*n* = 59) and HSV-2 (n = 5), Table 1. It was noteworthy that 15 cases who were only seropositive to IgG antibody were identified as CMV infection because the IgG antibody level sharply increased in sequential serum samples, and 8 cases were also positive to CMV-DNA test. 10 and 3 of the 12 CMV infections confirmed by PCR were seropositive to IgG and IgM antibodies, respectively. Meanwhile, 45 and 4 of the 47 women with CMV-DNA negative result were also seropositive to IgG and IgM antibodies, respectively. One HSV-2 infection confirmed by PCR was seropositive to only IgM antibody. Among the 4 PCR negative women for HSV-2, however, seropositive result (IgG antibody) was observed in only one case, Table 3.

**Table 3:** The serological results of CMV and HSV-2 for women with PCR test

Serologic results	Quantification of CMV-DNA		Quantification of HSV-2-DNA	
	Positive (*n* = 12)	Negative (*n* = 47)	Positive (*n* = 1)	Negative (*n* = 4)
IgM - IgG -	0	0	0	3
IgM + IgG -	2	2	1	0
IgM - IgG +	9	43	0	1
IgM + IgG +	1	2	0	0

## Discussion

The immune status and susceptible populations for TORCH pathogens should be evaluated by antepartum screening [7–9]. During the pregnancy, the TORCH infections at different status, i.e., primary infection of TOX, primary or recurrent infections of CMV, and primary infections at the first trimester for RV and at the third trimester for HSV, determine the risk of vertical transmission and severity of fetal [4, 5, 10]. However, the diagnosis of primary and recurrent TORCH infections by serological assay rely on a series of tests, such as the dynamic tests for both IgM and IgG antibodies to observe the seroconversion and change of IgG level, and an avidity test for the positive to IgG antibody [10, 11]. In this respect, TORCH screening should be started at right time (pre-gestation period, the first and third trimester), and not be considered as a single serum test. During the study period, more women in 2016 and 2017 than in 2015 performed TORCH screening due to the implementation of “two-child policy” in China since 1 Jan 2016 [12]. However, only 1.76% of the women performed dynamic TORCH tests and quite a few pre-gestation women (29.74%) ignored the screening test for IgG antibody in the study.

In the study, the seroprevalence of TORCH agents was significantly associated with BOH, but it was not effected by the age-specific distributions and periods of gestation. This was similar with other previous studies [13, 14]. RV, CMV and HSV-1 accounted for the majority of the TORCH infections with seropositivity of 90, 96.79 and 81.11% for IgG antibodies, however, the incidence of primary and acute TORCH infections was very low with seropositivity of 0.14–0.97% for IgM antibody. The findings are similar with previously published studies both in local [15] and elsewhere [16–18]. Owing to rarely eat raw meat in China, the seropositivity of IgG antibody for TOX in our study was lower than that in Turkey (31%) [16], India (28%) [17] and Canada (59.8%) [18]. In our study, the overall seropositvity of TOX, RV, CMV, HSV-1 and HSV-2 was 4.34, 90, 96.79, 81.11 and 6.1% for IgG antibodies. That implied only 4.34 and 6.10% of the child-bearing age women were immune to TOX and HSV-2 compared with 90% for RV, 96.79% for CMV and 81.11% for HSV-1.

The IgM antibody can be detected in different clinical situations, i.e., the primary TORCH infections, the persistence of IgM and the false positive due to Rheumatoid factor (RF) [10], Epstein-Barr (EB) virus [19] or antiphospholipid syndrome [20]. In the present study, among the 29 cases who were positive to IgM but negative to IgG antibodies in the first TORCH test, 6 cases had IgG seroconversion. In addition, of the 12 CMV infections and 47 CMV non-infections by PCR assay, the IgM antibody was detected in 2 and 4 cases, respectively. However, the negative to IgM antibody was observed in 15 CMV infections with dynamic increase for IgG level. The findings also demonstrate the opinion that the IgM result of a single serologic test is difficult and inconclusive to the diagnosis of TORCH infections [5, 21, 22].

## Conclusions

In summary, more attentions should be paid to TOX, CMV, HSV-2 and the women with BOH for TORCH screening in Xi’an region. Meanwhile, a greater emphasis needs to be placed on TORCH test used inappropriately in China, i.e., generally considered to be a single serum test, ignore the evaluation of pre-gestation immune status and dynamic monitoring of IgG antibody. For diagnosis of TORCH infection, the results of serial serologic tests, PCR and clinical manifestations should be taken into account.

## Data Availability

The data used in the study was permitted to be obtained from LIS by the Clinical Laboratory of the First Affiliated Hospital of Xi’an Jiaotong University. All patient information was conducted anonymously in the study.

## Abbreviations

BOH: Bad obstetric history
CFDA: China Food and Drug Administration
CMIA: Chemiluminesent Micropaticle Immuno Assay
CMV: Cytomegalovirus
EB: Epstein-Barr
HSV: Herpes simplex virus
LIS: Laboratory Information System
RF: Rheumatoid factor
RV: Rubella virus
TOX: *Toxoplasma gondii*
